# List context effects in languages with opaque and transparent orthographies: a challenge for models of reading

**DOI:** 10.3389/fpsyg.2014.01023

**Published:** 2014-09-15

**Authors:** Daniela Traficante, Cristina Burani

**Affiliations:** ^1^Department of Psychology, Catholic University of MilanMilan, Italy; ^2^NeuroMI, Milan Center for NeuroscienceMilan, Italy; ^3^Institute for Cognitive Sciences and TechnologiesISTC-CNR, Rome, Italy; ^4^Department of Life Sciences, University of TriesteTrieste, Italy

**Keywords:** reading aloud, list context effects, models of reading, strategic behavior, orthographic systems

## Abstract

This paper offers a review of data which show that reading is a flexible and dynamic process and that readers can exert strategic control over it. Two main hypotheses on the control of reading processes have been suggested: the route de-emphasis hypothesis and the time-criterion hypothesis. According to the former, the presence of irregular words in the list might lead to an attenuation of the non-lexical process, while the presence of non-words could trigger a de-emphasis of the lexical route. An alternative account is proposed by the time-criterion hypothesis whereby the reader sets a flexible deadline to initiate the response. According to the latter view, it is the average pronunciation difficulty of the items in the block that modulates the time-criterion for response. However, it is worth noting that the list composition has been shown to exert different effects in transparent compared to opaque orthographies, as the consistency of spelling-sound correspondences can influence the processing costs of the non-lexical pathway. In transparent orthographies, the non-lexical route is not resource demanding and can successfully contribute to the pronunciation of regular words, thus its de-emphasis could not be as useful/necessary as in opaque orthographies. The complex patterns of results from the literature on list context effects are a challenge for computational models of reading which face the problem of simulating strategic control over reading processes. Different proposals suggest a modification of parameter setting in the non-lexical route or the implementation of a new module aimed at focusing attention on the output of the more convenient pathway. Simulation data and an assessment of the models’ fit to the behavioral results are presented and discussed to shed light on the role of the cognitive system when reading aloud.

## INTRODUCTION

During the last decades, since the pioneeristic work of Coltheart (1978), several studies on word recognition have found that changes in the stimuli list context can influence latency and accuracy in different tasks. These results challenge the assumption that word recognition is an automatic process for skilled readers (Underwood, 1978); in contrast, they suggest that strategic components can alter word processing in relation to the composition of the list context. Moreover, data from different languages have revealed a complex pattern of results and suggested that the characteristics of the language system, in particular its orthography-to-phonology consistency, could be considered as a “macro-context” in which the system may develop its specific setting, with potential consequences on the suitability of different strategies in different languages.

The most widely accepted reading models offer a framework to simulate the processes involved in the recognition of a single item, but do not consider the list context in which that item is presented. This review is aimed at showing that the data on list context effects call for a new approach in reading modeling, in which additional components and/or mechanisms are to be included to take into account strategic behavior.

After a brief description of the dual-route cascaded model (DRC), of the parallel-distributed-processing model (PDP), and of the connectionist dual-process model (CDP), empirical data drawn from different languages will be presented in order to highlight the role that list context and language context can play in implementing different strategies when reading aloud.

The large number of experiments assessing strategic effects in different tasks, such as lexical decision or semantic categorization, are not considered in the present paper for two main reasons. Firstly, we aim at providing evidence for the activation of strategic behavior in one task, reading aloud, in which decision-level processes are not assumed to be involved. Thus, we intend to avoid possible confounds between strategies triggered by the list context composition and decisional strategies that are operating in tasks such as lexical decision or sematic categorization. Secondly, only one reading model (Harm and Seidenberg, 2004) implements semantic components, due to the high complexity of the model architecture required to take into account semantics. Accordingly, we thought it was more appropriate to consider only reading aloud studies, whose results can be simulated by means of the orthography-to-phonology mappings actually implemented by all the main computational models.

How data on list context effects may challenge the different modeling proposals and open new perspectives on the role of strategic control in reading aloud will be discussed in the final part of the paper.

## FROM PRINT TO SOUND: MODELS OF READING AND BENCHMARK EFFECTS

**FIGURE 1:**
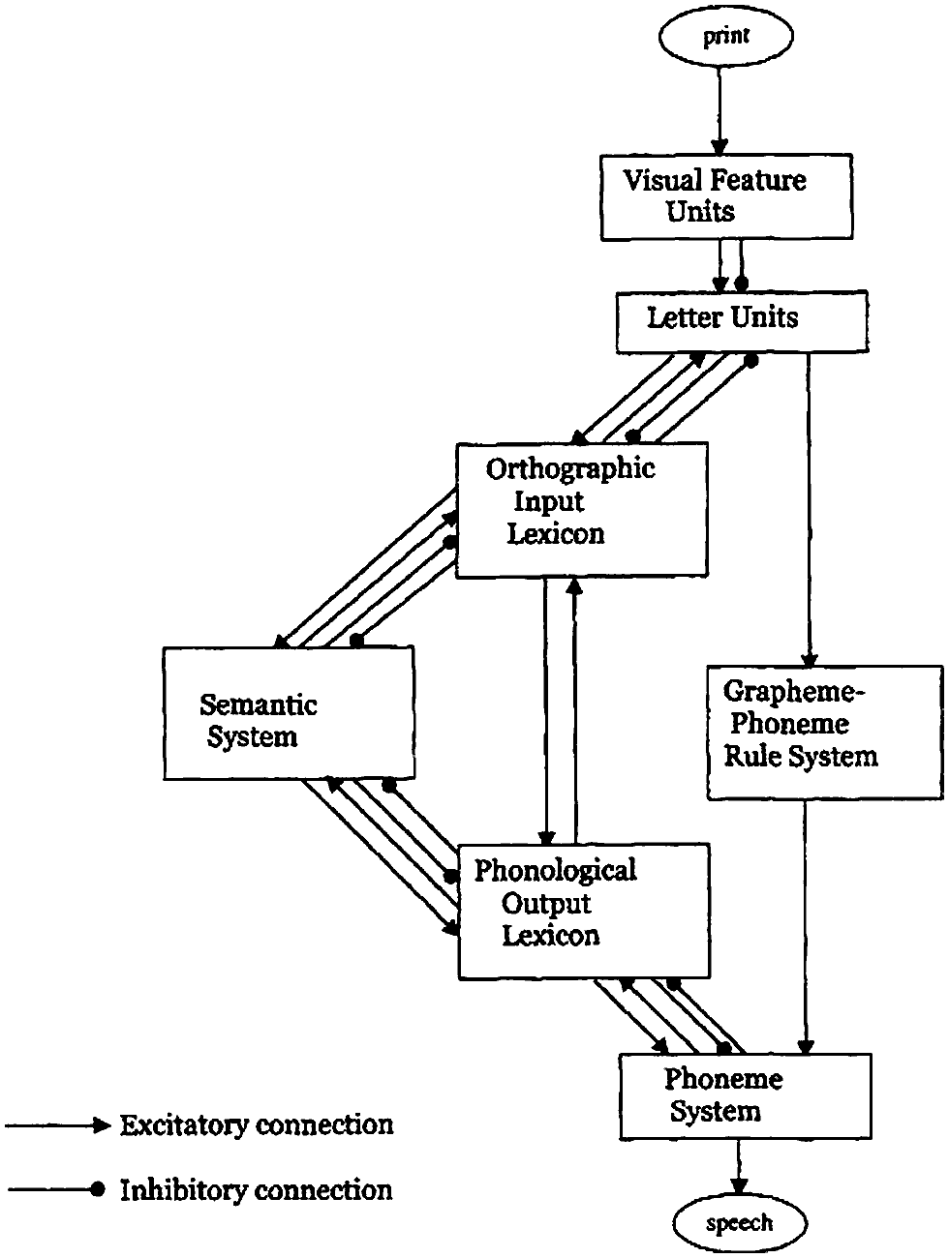
**The dual-route cascaded model.** From “DRC: a dual route cascaded model of visual word recognition and reading aloud” by Coltheart et al. (2001), *Psychol. Rev.* 108, p. 214. Copyright 2001 by the American Psychological Association.

The dual-route cascaded (DRC) model (Coltheart and Rastle, 1994; Coltheart et al., 2001) can be considered a computational evolution of the modeling tradition grounded in the 19th century modular approach. Despite its name, the model actually consists of three routes: the lexical semantic route, the lexical non-semantic route, and the grapheme-phoneme conversion (GPC) route (non-lexical route). However, the lexical semantic route has not been implemented yet (**Figure 1**). The model is cascaded because the activation is fed forward from one module to the following as soon as a process in that module starts, without waiting for the completion of the process itself.

The early modules from print to word recognition (visual feature units, letter units, orthographic input lexicon) form a three-layer network, working with interactive activation and inhibition among the layers. In the case of non-words, no lexical entry can be addressed, but it is possible to produce a phonological output through the grapheme-to-phoneme correspondence (GPC) route. This route starts operating after a series of cycles from the input onset and converts letters to phonemes from left to right, serially, according to rules set on statistical grounds (Rastle and Coltheart, 1999). The generated phonemes add activation to units in the phoneme system, a layer common to both the lexical and non-lexical routes, in order to produce letter string pronunciation. However, non-words are not only read through the non-lexical route because they partially activate word neighbors^1^ in the orthographic lexicon and these word units feed-forward activation to the phonological representations and to the phoneme system.

The need for implementing two different routes to read words and non-words has been challenged by the parallel-distributed-processing (PDP) model (Plaut et al., 1996). This is a one-route model of reading aloud, whose architecture is a three-layer network trained by an error-minimization learning algorithm. In the PDP model, all letter strings (both words and non-words) activate phonemic units in parallel. The distributional features of the input corpus are represented in the activation patterns within and between orthographical and phonological layers and all spelling-sound mappings depend on the parameter setting in the intermediate layer (hidden units). In this architecture, there are no specific pathways for reading words and non-words: “The information concerning spelling-sound correspondences, derived from exposure to actual words and encoded by the weights in such networks, is also used in generating pronunciations for unfamiliar stimuli” (Seidenberg et al., 1994, p. 1178).

**FIGURE 2:**
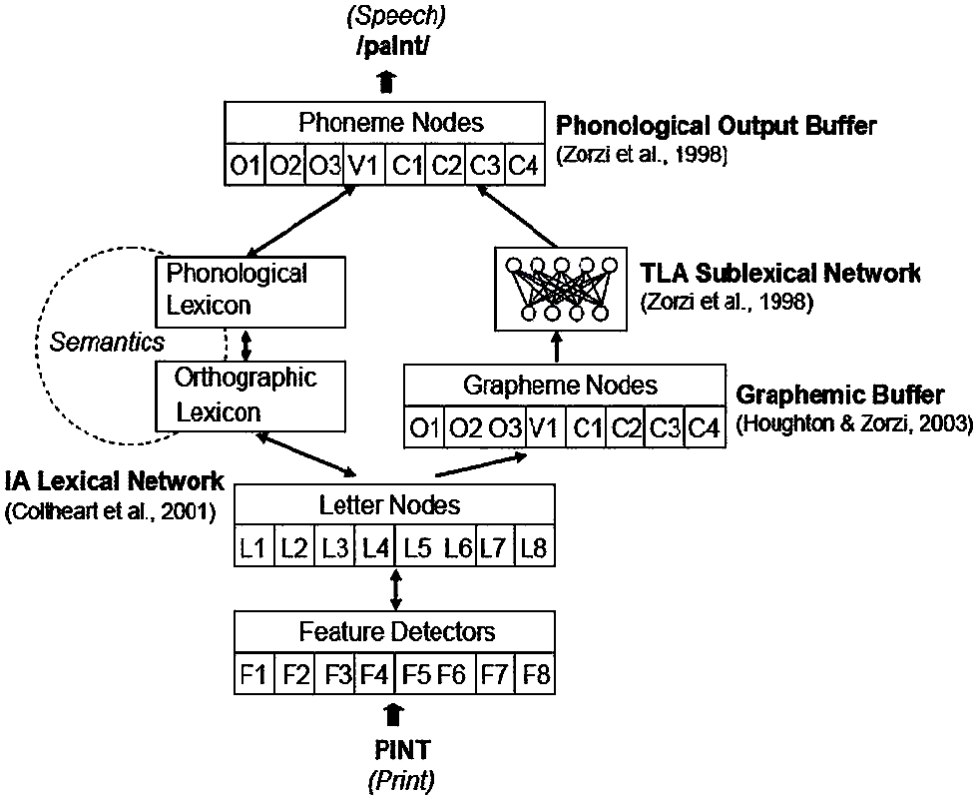
**The connectionist dual process model (CDP+).** O = onset; V = vowel; C = coda; TLA = two-layer assembly; IA = interactive activation; L = letter; F = feature. From “Nested incremental modeling in the development of computational theories: the CDP+ model of reading aloud” by Perry et al. (2007), *Psychol. Rev.* 114, p. 280. Copyright 2007 by the American Psychological Association.

The connectionist dual-process (CDP) model developed by Zorzi and colleagues (CDP: Zorzi et al., 1998; CDP+: Perry et al., 2007; CDP++: Perry et al., 2010) builds on the existing PDP and DRC models by combining features of both and is aimed at overcoming their limits. In the CDP model (**Figure 2**), spelling-sound connections are implemented, in parallel, *via* two pathways: a print-to-sound mapping mediated by lexical representations, implemented through a localist lexical route based on the interactive activation model as in Coltheart et al. (2001); a direct mapping from graphemic to phonemic units, implemented through a connectionist network (TLA: two-layer assembly model) as in Zorzi et al. (1998). This choice allows the CDP model to have not only an efficient solution to simulate lexical access in word reading, as in the DRC, but also a network for assembled phonology, that overcomes the absence of a learning mechanism in the DRC, a model which is fully hardwired and whose non-lexical route works according to partially hand-coded sets of grapheme-to-phoneme conversion rules. Due to this network, the CDP model is able to simulate reading acquisition and developmental readingdisorders, similar to the PDP model.

Pritchard et al. (2012) tested the DRC and CDP models in reading non-words comparing their performances to human responses. The DRC model showed a better match to participants’ pronunciations (matching rates: 73.5% for DRC vs. 37.6% for CDP++). However, unlike behavioral data, the DRC model did not produce any lexicalization, while the CDP++ model produced very high lexicalization rates. In a recent paper, Perry et al. (2014) assessed the fit of DRC and CDP++ to the behavioral data in French, an orthography in which there are silent consonants at the end of words. The authors found that human readers, in reading non-words, tended to pronounce silent consonants that are not phonologically transcoded when detected in words. The DRC model, with the implementation of grapheme-to-phoneme rules for French, produced the pronunciation of these consonants in only 5.8% of the trials, while human readers pronounced them in 57.8% of the trials. The CDP++ model reached a rate of 41.2% pronunciations of silent consonants and this result was obtained through “a sublexical plus lexical analogy mechanism” (Perry et al., 2014).

Computational models test their claims to adequacy by simulating basic phenomena observed in reading aloud, that can be considered *benchmark effects* (Coltheart et al., 2001). In the present work only the benchmark effects, which have been proved to be influenced by stimulus list context will be presented: the lexicality effect, the length and length *by* lexicality effects, the word frequency effect, the regularity and regularity *by* frequency effects.

The *lexicality effect* (i.e., the observation that reading words is faster than reading non-words), in a lexicon-and-rule model like the DRC, is referred to the activation of different routes and modules, in relation to the lexical status of the stimulus. A non-word like BONT can activate some orthographic neighbors like FONT, BENT, BOND in the lexicon and gain activation in the phonemic system from these neighbors, but can be pronounced correctly only through a sequential activation of the phonemes corresponding to the graphemes B-O-N-T.

On the other hand, a regular item like WORD is likely to gain activation in the phonemic system from both routes, as both its lexical representation and the GPC rules produce a coherent phonemic pattern, which leads to a fast and correct response (Yates, 2010). In DRC and in CDP models, direct access to lexical representations, triggered by words, is faster than the serial application of GPC rules adopted in reading non-words, and this mechanism can explain why words are read faster than non-words. The PDP model offers an explanation in terms of frequency of activation of phonological patterns involved in the pronunciation of the target, assuming that non-words activate more rare orthographic-phonemic associations than words.

The serial processing of grapheme-to-phoneme conversion through the non-lexical route is also considered the mechanism that gives rise to the *length effect* (i.e., the longer the string of letters, the slower the reading latency). Overall, this effect is strong in reading non-words, while it is not found consistently in word reading (*length by lexicality effect*). While dual-route models can explain these effects quite well, as they assume that the sequential procedures involved in the grapheme-to-phoneme mapping are more time-costly than the direct access to lexical representations, the length *by* lexicality effect is particularly challenging for the PDP model. In fact, this model provides an account for the additional motor programming required by longer strings, but offers no ground for expecting differences in the visuo-perceptual scanning of words and non-words.

The *word frequency effect* (i.e., high frequency words are read faster and more accurately than low frequency words) is considered evidence for the activation of representations (either localist or distributed) in the orthographic lexicon (or system). All models assume that the speed of this activation is a function of the frequency of use of the corresponding words in the written language. Thus, lexical representations of high frequency words are activated faster than lexical representations of low frequency words.

Some interesting effects have been observed in reading exception words, like PINT or YACHT. These words are usually read more slowly than regular words such as FOND (*regularity effect*), but this effect is reliable only for low-frequency words (*regularity by frequency effect*). This phenomenon has been interpreted by the dual-route models as the result of an interference, in the phonemic system, between the output of the phonological lexicon and that of the grapheme-to-phoneme mapping mechanisms, which are doomed to fail. The PDP model refers this effect to the low level of activation of the phonological patterns involved in the pronunciation of low frequency exception words, but this model has some difficulties in simulating the pronunciation of a few low-frequency irregular words (e.g., AISLE). For this reason, Seidenberg et al. (1994) proposed that low-frequency irregular words can be read through the semantic system, the third component of the so-called triangular model, not implemented by Seidenberg and McClelland (1989), but implemented in the model of Harm and Seidenberg (2004).

The ability to simulate the above mentioned benchmark effects has been considered a validity test for the computational reading models. However, it is worth noting that these effects are not found consistently in the behavioral data, as they can be influenced by list composition. In fact, the presence in the stimuli list of either words and non-words mixed together (*mixed context*) or of only one type of stimuli (only words or non-words: *pure context*) can alter the size of those effects. Moreover, data from different language contexts offer a complex picture with inconsistent results.

In the following sections, a review of some seminal works will be presented that can be considered representative contributions to the debate on the mechanisms underlying reading aloud in different list and language contexts (**Table 1**). In the section beneath, studies focusing on the issue of “which” pathway is mostly involved in different list contexts will be described, and the so-called *route de-emphasis* and *time-criterion* hypotheses will be introduced. The role of the consistency of the orthography-to-phonology correspondence will be discussed in the subsequent section, in which data on list context effects from both opaque and transparent orthographies will be presented.

**Table 1:** Pathway control and time criterion setting in reading processes: evidence accounted for in the present review.

Language	Reference	Main results	Suggestions for modeling
English	Monsell et al. (1992)	*High-frequency exception words*: delayed in mixed list with non-words; *Low-frequency exception words*: not delayed in mixed list with non-words; *Non-words*: delayed in mixed list with low-frequency words. No effects with high-frequency exception words.	In mixed lists with exception words, non-lexical route is inhibited (*route de-emphasis hypothesis*)
	Rastle and Coltheart (1999)	Regularity effect size is reduced when fillers are high-frequency exception words with irregular phoneme in the first position.	In reading exception words, the inconsistency between lexical and non-lexical routes triggers a general slowing of non-lexical route (*route de-emphasis hypothesis*)
	Zevin and Balota (2000)	*Low-frequency exception words*: more regularization errors when primed by non-words than by exception words; *Non-words*: slowed-down when primed by low-exception words	A separate control mechanism which computes the conflict between lexical and non-lexical route, and can slow down the non-lexical route in the case of exception words (*route de-emphasis hypothesis*)
	Reynolds and Besner (2008)	*Exception-words*: switch costs arise when exception word follows non-word *Low-frequency regular words*: switch costs neither after high-frequency exception words nor after non-words.	Separate control mechanism that modulates reading process on the basis of previous trials (*exogenous control*)
	
	Lupker et al. (1997)	*High-frequency exception words*: delayed in mixed list with non-words; *Low-frequency exception words*: faster RTs (but lower accuracy) in mixed list with non-words than in pure list; *Non-words*: faster RTs (but lower accuracy) in mixed list with high-frequency exception words and regular words than in pure list; *Regular words*: faster RTs in pure than in mixed list	Readers would set a time criterion to start articulation, according to the difficulty of the stimuli. In mixed list the criterion is beyond the preferred responding point for the fast stimuli but prior to the preferred responding point for the slow stimuli (*time-criterion hypothesis*)
	Kinoshita and Lupker (2003)	*Regularity effect* was not affected by prime lexicality (either low-frequency exception words or non-words); *Frequency effect* was reduced when words follow fast non-words; *Lexicality effect* was reduced when prime were (both slow and fast) non-words	*Lexical checking* is applied after a phonological code has been produced, in order to assess the matching between the codes generated by the two routes. The massive presence of non-words would lead to skip this check

Persian	Baluch and Besner (1991)	Transparent words: frequency and semantic priming effects only in pure context.	Decision mechanisms which selects the output from the routine (lexical or non-lexical) that first makes a response viable. In the case of the presence of non-words, only the non-lexical route is considered
Italian and English	Tabossi and Laghi (1992)	Italian: semantic priming in naming words only in pure list and not in mixed list. English: semantic priming in both pure and mixed context	In a *transparent orthography* the use of either the routes in reading words is influenced by the list context; in an *opaque orthography*, the use of lexical route in reading words is mandatory
Turkish	Raman et al. (2004)	Using non-words matched to words in reading speed, the *frequency effect* is always reliable	Also in a *transparent orthography* words are read through the lexical route, even though in mixed context with non-words Data on latency support the *time-criterion hypothesis*
Italian	Pagliuca et al. (2008)	*Lexicality effect* is reliable in both pure and mixed context; *Words* are read faster in the pure than in the mixed condition; *Non-words* are not influenced by the list context	Lexical route is never completely shut down, but is the main route to read words also in a transparent orthography; Data on non-words do not support the time-criterion hypothesis
Italian	Paizi et al. (2010)	Words: *frequency effect* reliable in both pure and mixed context; *Length effect* reliable only in mixed condition; Non-words: *length effect* reliable in all conditions	Data inconsistent with both de-emphasis and time-criterion hypotheses; Italian readers cannot block the activation of the lexical route, but have no reason for shutting down the non-lexical route, as it is not resource demanding in a transparent orthography like Italian

In the final section, findings from the literature on the role of stimulus quality and proportion of related primes and targets in modulating frequency effects will offer further suggestions on the relation between list context effects and “how” the reading processes unfold. Proposals for new computational approaches, based on dynamic adaptation to the context conditions and trial history, will be presented and discussed, as they are likely to become the framework for research on reading processes in the next future.

## “WHICH” PATHWAY FROM PRINT TO SOUND? ROUTE DE-EMPHASIS vs. TIME-CRITERION HYPOTHESIS

### THE ROUTE DE-EMPHASIS HYPOTHESIS

**FIGURE 3:**
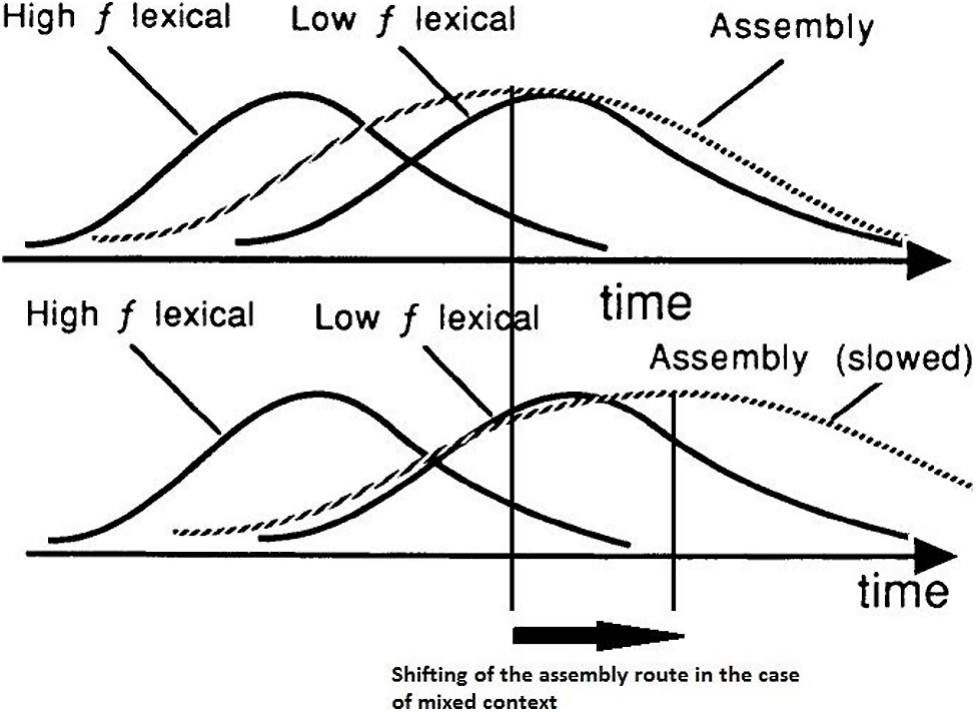
**Top:** imaginary distributions of processing time for high- and low-frequency exception words (*High f lexical*, *Low f lexical*, respectively; solid curves) and for non-words (sublexical *assembly* process; broken curve). **Bottom:** the same, with the distribution for the assembly process shifted to the right, to simulate the hypothesized effect of trying to ignore assembled output. Adapted from “Lexical and sublexical translation of spelling to sound: strategic anticipation of lexical status” by Monsell et al. (1992), *J. Exp. Psychol. Learn. Mem. Cogn.* 18, p. 463. Copyright 1992 by the American Psychological Association.

Monsell et al. (1992) tested the strategic dissociation of lexical and non-lexical routes in English-speaking readers, focusing their attention on latency and accuracy when reading aloud non-words and exception words with the former assumed to be processed through GPC rules, the latter through the lexical pathway. In their experiment, participants had to read exception words and non-words, either in pure or in mixed blocks. Within word blocks, the frequency of use was blocked too, as the items of each block were all high frequency words or low frequency words. They found that reading high-frequency (very familiar) exception words was delayed by the presence of non-words in the list (mixed block), in comparison to the latencies observed in pure lists; however, when the participants expected to see low-frequency (less common) exception words, reading was not delayed by the presence of non-words in the list. On the other hand, reading of non-words was delayed, in comparison to a pure list condition in which only non-words were presented, by the presence of low-frequency exception words and not by the presence of high-frequency exception words. The authors proposed an explanation of these effects grounded on the distributions of processing times for the lexical and the non-lexical processes (**Figure 3**).

They made the assumption that, in the case of pure lists, the distribution of processing times for non-words has a large overlap with the distribution of processing times for low-frequency exception words, while the overlap with the distribution of high-frequency exception words is smaller (**Figure 3**: top). The non-lexical process should be slowed down in the case of mixed lists with low-frequency words, because the reader has to ignore the non-lexical output to increase the probability of a correct pronunciation of the exception words (**Figure 3**: bottom). On the side of low-frequency exception words, though, the slowing down of non-word processing does not have much effect, as the two distributions significantly overlap in any case, both in pure and in mixed conditions. As for high-frequency exception words, the expectancy of all exception words, as in a pure list, slows down the non-lexical process as well, and leads to faster RTs than in a mixed list as the spread between the two distributions increases. Monsell et al. (1992) proposed a continuous integration model of reading suggesting that “a phonological description is built up incrementally using fragments of information transmitted asynchronously from both processes” (p. 464). When information coming from the two processes is congruent, as in the case of regular words, the articulation of the currently available phonological description begins faster than when it is conflicting, as in the case of exception words. In the latter case, skilled readers can apply selective inhibition of (or inattention to) the non-lexical route, with different effects on latency distributions for high- and low-frequency exception words, as described above.

**FIGURE 4:**
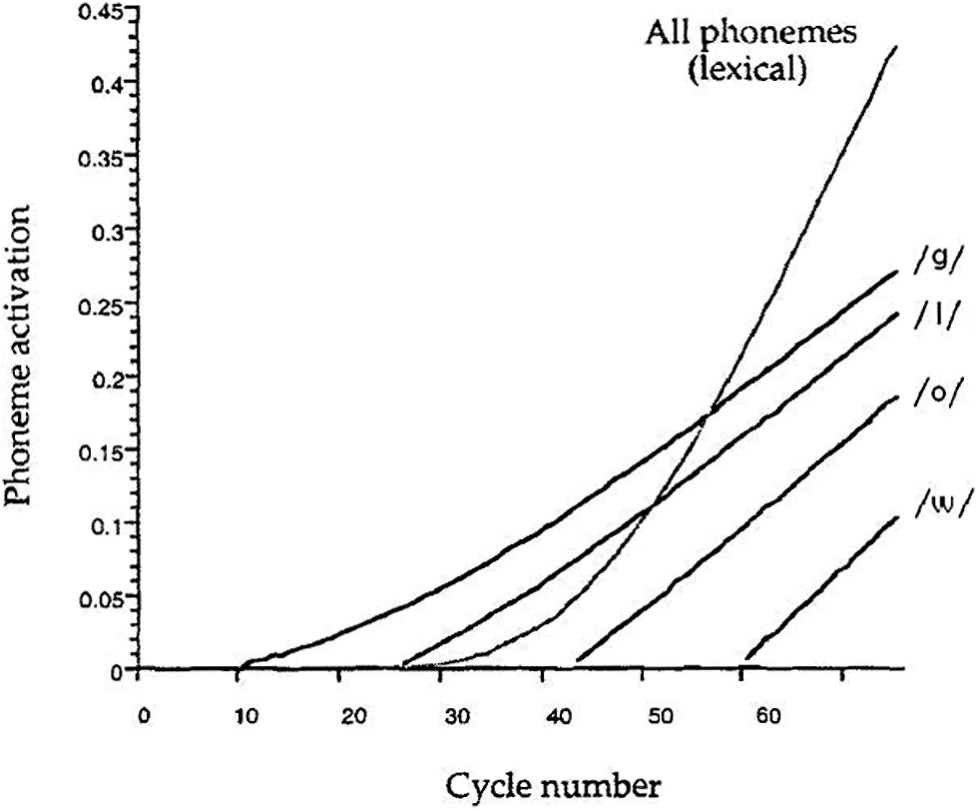
**Lexical and non-lexical activation of the phonemes of the exception word GLOW during its reading by the DRC model.** Adapted from “DRC: a dual route cascaded model of visual word recognition and reading aloud” by Coltheart et al. (2001), *Psychol. Rev.* 108, p. 234. Copyright 2001 by the American Psychological Association.

The effects of the presence of exception words in the experimental list on reading performance have been challenged by Coltheart and Rastle (1994). The authors aimed at assessing the strategic control operated by readers on the use of the lexical and non-lexical routes, by inserting different types of filler items in the naming experiments. They assumed that the presence of non-words should favor the use of the non-lexical route, while high-frequency exception words are expected to favor the use of the lexical route. The regularity effect, interpreted as an interference of the non-lexical route on lexical processing in reading exception words, should be larger when fillers are non-words than when they are high-frequency exception words, as the latter condition should induce neglect of the non-lexical route. Coltheart and Rastle’s (1994) study did not confirm this hypothesis. However, in a further study, using only exception words with the irregular phoneme in the first position, Rastle and Coltheart (1999) found the expected list context effect. This means that a general slowing of the non-lexical route is triggered only when the inconsistency between the lexical and non-lexical routes already occurs at the beginning of the process (e.g., for words like “chef”); however, if the irregularity is in the middle of the exception words (e.g., “glow”), their lexical representation is accessed before the sequential letter-by-letter activation could interfere with lexical processing (**Figure 4**). These data are consistent with Monsell et al.’s (1992) findings, and support the route de-emphasis hypothesis.

This hypothesis has been implemented by modifying the parameters controlling the activation of the non-lexical route. Rastle and Coltheart (1999) successfully simulated the delaying effect of exception word fillers on naming regular word and non-word targets by increasing the number of cycles (from 17 to 22) elapsed before the non-lexical route can process the next letter. Perry et al. (2007) proposed a similar parameter manipulation, increasing the number of cycles occurring between the processing of each letter in the non-lexical pathway of CDP+ from 15 to 17. They obtained a delay in reading non-words (7.94 cycles) and words (2.94 cycles) proportional to the delay in behavioral data (20 and 12 ms, respectively: Rastle and Coltheart, 1999, p. 494, Table 5). In contrast, the modification to the DRC made by Rastle and Coltheart (1999) led to an overestimation of the delay for non-words (22.49 cycles) and an underestimation for words (0.56 cycles; p. 495, Table 6).

Zevin and Balota (2000), in order to account for the dependence of reading on specific sources of information, used a priming procedure in which each trial consisted of five primes followed by a target and all the stimuli had to be read aloud. This procedure was aimed at creating “a situation in which dependence on the most efficient pathway for processing the prime stimuli would be maximally beneficial” (p. 123). Their results showed that non-word naming is slowed down after naming a sequence of (five) low-frequency exception word primes – a condition in which the non-lexical route is de-emphasized. Moreover, they found that low-frequency exception words gave rise to more regularization errors when primed by a sequence of (five) non-words than by other exception words, as the route de-emphasis hypothesis predicts.

In order to simulate the route de-emphasis effects, Zevin and Balota (2000) proposed adding a separate control mechanism to the DRC model which obtains information from the phoneme system and computes “the conflict or the ratio of contributions between the lexical and sublexical routes on a given trial” (Zevin and Balota, 2000; p. 132). This control system can slow down the sublexical route, as proposed by Rastle and Coltheart (1999) in the case of exception word primes, or change strategy, by gaining from the outputs of both routes, in the case of non-word primes, because “… readers are sensitive to the processing demands presented by different stimuli in a word-naming task and […] they are able to adjust their dependence on different sources of information accordingly” (Zevin and Balota, 2000; p. 133). The same mechanisms might in principle apply also to a PDP model to adjust the relative contribution of the direct orthography-to-phonology network and of the semantic system in spelling-to-sound translation.

However, it is an open question whether the changes in strategy are triggered by the features of the item itself (*exogenous control*) or by the reader’s expectations (*endogenous control*), developed on the basis of the trial sequence. Some suggestions on this issue come from studies carried out with the task-switching paradigm. Reynolds and Besner (2008), in studying the use of the reading routes, adopted the alternating runs paradigm which consists of presenting participants with two tasks in a predictable AABB sequence. With this paradigm, RTs on switch trials (A→B) are usually slower than on stay trials (A→A). The switch costs are interpreted as the output of an exogenous control component of the process, driven by the presentation of the task-relevant stimulus.

For instance, when the A task consists of reading high-frequency exception words and the B task in reading non-words (Reynolds and Besner, 2008: Experiments 1–3), switch costs arise from the interference between the lexical strategy and the non-lexical strategy, caused by the presentation of a non-word. The same happens when an exception word follows a non-word. Switch costs were neither found when low-frequency regular words were presented after high-frequency exception words (Experiment 4) nor when the same regular words were in an alternating sequence with non-words (Experiment 5). These results seem to confirm that low-frequency words are likely to be read through the lexical route when mixed with high-frequency exception words and through the non-lexical route when presented with non-words. The switch costs are consistent with the proposal of a separate control mechanism that modulates reading process on the basis of previous trials.

### THE TIME-CRITERION HYPOTHESIS

A different perspective has been offered by Lupker et al. (1997), Kinoshita and Lupker (2002, 2003), Chateau and Lupker (2003). In keeping with Monsell et al.’s (1992) results, Lupker et al. (1997) found that, in English, both high-frequency exception words and regular words were read faster in a pure than in a mixed condition. But, in contrast with Monsell et al.’s (1992) results, non-words were named faster when mixed with words (both high-frequency exception words and regular words) than in a blocked condition. Moreover, low-frequency exception words achieved faster RTs in a mixed condition with non-words. The data on low-frequency words and on non-words, however, showed that the gain in RTs occurs at the expense of accuracy, as there was a trade-off between latencies and accuracy for these stimuli.

In order to interpret their results, Lupker et al. (1997) proposed the *time-criterion hypothesis*: readers would set a time criterion to start articulation, which is determined by the difficulty of the stimuli and is aimed at maintaining an acceptable level of accuracy and rapidity. In this way, when easy (regular words, high-frequency exception words) and difficult (non-words, low-frequency exception words) stimuli are mixed together, “the criterion would have tended to stabilize at a point that was beyond the preferred responding point for the fast stimuli but prior to the preferred responding point for the slow stimuli” (Lupker et al., 1997; p. 578). This claim should also explain the trade-off between latencies and errors: the early start of the articulation of a difficult stimulus can lead to a lower level of accuracy. According to the authors, the criterion can also be set in relation to the task and to the experimental procedure, since stressing speed or accuracy can produce different effects.

Kinoshita and Lupker (2003) adopted the priming paradigm introduced by Zevin and Balota (2000), but selected three types of primes: fast non-words (short and with high N-size), slow non-words (long and with low N-size) and low frequency exception words. They studied the influence of prime lexicality (words/non-words) and of prime type (slow vs. fast stimuli) on the size of three benchmark effects, namely the regularity effect, the frequency effect and the lexicality effect. The regularity effect was significant and it was not affected by the prime lexicality (words/non-words). However, the reading latencies of the targets were affected by the prime type, as targets were named faster when following faster primes, irrespective of being exception words or (fast) non-words (Experiment 1). The frequency effect was reduced in the case of a fast non-word context, but not in the case of a slow non-word context. The authors interpreted this result as evidence that a context composed of rapidly named stimuli reduces the difference between high and low frequency words, because of a floor effect for high frequency words (Experiment 2). While the results of the first two experiments were in line with the time-criterion hypothesis, the third experiment showed inconsistent data. In fact, the lexicality effect was reduced in the case of non-word (both fast and slow) primes. The authors interpreted this result as a consequence of the application of a second – lexical checking – reading strategy, according to which “prior to emitting a naming response, readers have the option of consulting the phonological output lexicon in order to determine whether the code generated by the phonological coding process matches a code in the output lexicon” (Kinoshita and Lupker, 2003; p. 412). Lexical checking would take place after a phonological code has been produced and can be skipped in the case of a massive presence of non-word stimuli.

The complex pattern of results described above offers a view of reading as a dynamic process, in which different procedures for obtaining phonology from print (the lexical or non-lexical pathways) can be strategically activated according to the characteristics of the list context. In the following section, studies will be reported aimed at assessing whether the consistency of grapheme-to-phoneme correspondence may introduce further differences among languages in the way in which strategic control is applied. In fact, a fully consistent orthography could make it possible, in principle, to read words through the only involvement of the non-lexical route and this opportunity might give rise to a completely different pattern of list context effects, in comparison to opaque orthographies. Data from neuroimaging studies support the view of different reading processes in English, that has an opaque orthography, as opposed to Italian, whose orthography is considered to be transparent (Paulesu et al., 2000). Also the psycholinguistic grain size theory (Ziegler and Goswami, 2005, 2006; Goswami and Ziegler, 2006) pointed out that children of transparent orthographies would learn reading by relying on small units of phonological recoding, while children of opaque orthographies are supposed to use multiple phonological recoding strategies, based on larger units, to avoid mispronunciations. It can thus be assumed that differences in the early phases of learning to read might produce different reading behaviors in the mature system.

## EVIDENCE FROM DIFFERENT LANGUAGES: THE ROLE OF ORTHOGRAPHIC CONSISTENCY

To shed light on the two main perspectives described above (de-emphasis hypothesis and time-criterion hypothesis), in the context of a transparent orthography like Turkish, Raman et al. (2004) followed Kinoshita and Lupker’s (2003) approach. They manipulated non-word length, in order to create two lists of non-words that were matched on reading time (rather than length) to high-frequency and low-frequency words, respectively. Thus they obtained four different lists of stimuli: (a) high-frequency words and (b) corresponding fast non-words, (c) low-frequency words and (d) corresponding slow non-words. By using non-words matched to words in reading speed, the authors could test, separately, the effect of the time-criterion induced by the reading time of the stimulus context and the effect of shifting from the lexical to the non-lexical route in the presence of non-word stimuli. To assess the involvement of the lexical route, the authors analyzed the size of the word frequency effect in different list contexts, assuming that, following the de-emphasis hypothesis, the frequency effect should not be reliable in mixed condition (words and non-words together), in spite of its reliability in pure (only words) condition.

Contrary to the latter prediction, Raman et al. (2004) found that the frequency effect was significant in all conditions, even though its size was modulated by list composition. The pattern of results shows that, also in a transparent orthography, words are read through the lexical route as the frequency effect is reliable in all conditions irrespective of the presence of non-words in the list. Moreover, the naming latencies for high-frequency words are influenced by the mean difficulty of the list thus supporting the time-criterion hypothesis. Accordingly, the authors claimed that “it appears that neither the lexical nor the non-lexical route is under strategic control of Turkish readers and that the data are best explained by a time criterion position” (Raman et al., 2004; p. 498).

This conclusion is not consistent with previous results in other languages with transparent orthographies, like Persian (Baluch and Besner, 1991) and Italian (Tabossi and Laghi, 1992). In fact, in those studies the presence of non-words eliminated lexical and semantic effects in reading words. In particular, Baluch and Besner (1991) found that Persian transparent words (i.e., with printed vowels) are named by using the non-lexical route (as indicated by the absence of semantic and frequency effects) when presented in a mixed context with non-words, while they are likely to be read by means of the lexical route (indicated by the presence of semantic and frequency effects) when presented in a pure context. To explain these results, the authors proposed a decision mechanism which selects the output from the route (lexical vs. non-lexical) that first makes a response available. In the case of the presence of non-words, such a mechanism would first consider the output of the non-lexical route, thus eliminating lexical and semantic effects in transparent word reading. In the case of words alone, only the lexical route would be selected. Raman et al. (2004) interpreted the lack of significance of frequency effect in the study by Baluch and Besner as due to the use of non-words matched to the words in length and not in reading speed. Such non-words are likely to be so difficult to lead the time criterion at a very high level, slowing down high-frequency words to such an extent that the frequency effect is eliminated.

However, Tabossi and Laghi (1992), for the Italian language, also came to a conclusion consistent with Baluch and Besner’s (1991) results. They adopted the same experimental design to assess list context effects in two orthographies with different degrees of spelling-sound consistency: Italian and English. The authors found different list context effects for the two languages. For Italian, semantic priming in naming words occurred only in a pure context (words alone), while in a mixed context, in which both words and non-words were presented, semantic priming was not significant. These data suggest that when non-words are present, Italian words are likely to be read through the non-lexical route. In contrast, in English, semantic effects were found not only in pure, but also in mixed contexts. The results indicate that in reading a language with an opaque orthography lexical access is mandatory.

It is worth noting, however, that both in Baluch and Besner’s (1991) and in Tabossi and Laghi’s (1992) studies, some evidence for the activation of the lexical pathway in reading words was also found in a mixed context. In Persian, the lexicality effect emerged in all list contexts, with transparent words being read faster than non-words in both pure and mixed lists. This result shows that words, differently from non-words, can gain activation not only from the non-lexical route, but also from the phonological output lexicon, which feeds forward to the phonemic output buffer and makes word naming faster than non-word naming. Overall, this study shows the high flexibility of the word-naming process in a transparent orthography like Persian.

Similarly, Tabossi and Laghi (1992) demonstrated that it is possible to obtain semantic effects also in Italian, in a mixed context, by adding to the list of stimuli a small proportion (about 20%) of trisyllabic words stressed on the first syllable (e.g., *fàcile*, easy). In order to correctly name these words, Italian readers have to access lexical knowledge, while reading them through the non-lexical route is likely to lead to the default stress assignment (valid for about 70% of Italian words) on the penultimate syllable (e.g., *facìle*^∗^), that produces a wrong response. In this condition, the authors found effects of semantic priming in Italian, just as in English. These data led the authors to conclude that skilled readers rely on their lexical knowledge in naming most common words, regardless of the different writing systems. Only in unusual conditions, in which they read lists of non-words and regular words, readers of transparent orthographies can find it more useful to apply the non-lexical assembled phonology. Switching from lexical reading to the unusual non-lexical route is a matter of strategy that educated adults can apply even if they may be unaware of this.

Several years later, Pagliuca et al. (2008) came to similar conclusions. They tested list context effects on word and non-word reading in Italian, contrasting the de-emphasis and time-criterion hypotheses. They presented readers with high-frequency and low-frequency Italian words in pure and mixed conditions with non-words. Words in the pure condition were read faster than in the mixed condition and this evidence is consistent with both the route de-emphasis and the time-criterion hypothesis. In contrast, reading non-words was not influenced by the list context at all and this result cannot be accounted for by the time-criterion hypothesis. Furthermore, the authors found that, also in a transparent orthography like Italian, the lexicality effect is reliable in all conditions (pure and mixed), even when non-words are compared to low-frequency words. Pagliuca et al. (2008) concluded that “these data support the view that the lexical route is never completely shut down but is instead the main route used in naming words, regardless of orthography depth” (Pagliuca et al., 2008; p. 431).

Strong support for the use of the lexical pathway in a transparent orthography comes from further research conducted in Italian, in which the authors (Paizi et al., 2010) adopted an experimental design similar to Raman et al. (2004). The main difference was that non-words were not matched to words on reading times, but on length in letters, N-size, bigram frequency, orthographic rules, and initial phoneme. Paizi et al. (2010) also tested the effect of stimulus length, as the role of length in reading low-frequency words and non-words could be ascribed to the use of the non-lexical route. They found that the frequency effect was reliable in all conditions, even when words were mixed with non-words. These data are not consistent with previous research that found for transparent-orthography languages the reduction (Raman et al., 2004) or the disappearance (Baluch and Besner, 1991; Tabossi and Laghi, 1992) of the frequency effect in mixed conditions. In contrast to the stability of the frequency effect in all list conditions, the effect of length for words was fully significant only in the all-mixed condition in which high frequency and low frequency words were presented mixed with each other and to their corresponding non-words. For non-words the effect of length was fully reliable in all conditions. Hence, Paizi et al.’s (2010) data do not support the route de-emphasis account, as the reliability of the frequency effect in all conditions calls for a constant involvement of lexical activation, irrespective of the presence of non-words in the list. However, these data do not support the time-criterion account either, as there are no relevant differences across conditions for any kind of stimuli. The authors proposed that for Italian readers it is impossible to block the activation of the lexical route (as suggested by the persistence of the frequency effect). However, Italian readers also have no reason for shutting down the non-lexical route when reading words (as indicated by the varying length effect for words in the presence of a constant length effect for non-words), because, due to the ease of applying rules of print-to-sound conversion, the non-lexical route in Italian is not resource demanding. This latter interpretation also applies to the absence of any influence of list context in non-word reading reported by Pagliuca et al. (2008).

## OPEN QUESTIONS AND NEW APPROACHES: DOES LIST CONTEXT AFFECT “HOW” PROCESSING UNFOLDS?

The complexity of the results summarized above indicates that current theories and models are far from providing an adequate understanding of the mechanisms actually involved in reading processes. O’Malley and Besner (2008) claimed that one of the limits of the main computational approaches in this field is the assumption of cascaded activation as a fix processing mode. The authors suggested that the experimental context has an influence not only on *what/which* pathway is involved or slowed down, but also on *how* processing unfolds over time, i.e., whether the mechanisms that rule the functioning of the system may change and *why*, in case a change is triggered. In other words, they proposed that the list context may lead to modifications in the modality in which the decoding process is implemented and this change is detectable only by considering joint effects of different variables, which tap into different processing steps.

O’Malley and Besner (2008) offered evidence in favor of their view by jointly analyzing and modeling the effects of stimulus quality and frequency. They started from the observation that evidence from the lexical decision task shows additive effects of stimulus quality and frequency on RTs (Stanners et al., 1975; O’Malley et al., 2007; Yap and Balota, 2007), while data on reading aloud support interactive effects between the two variables (O’Malley et al., 2007; Yap and Balota, 2007). They demonstrated, in a set of three experiments, that the inconsistency between the results in the two tasks is not due to the task itself, but to the presence of non-words in the lexical decision procedure and their absence in the reading aloud experiments. In fact, asking participants to read aloud words and non-words in a mixed list, they obtained the same additive effect observed in lexical decision, while data from reading aloud a pure list of words showed interactive effects.

The authors, in the framework of the DRC model, advanced the *lexicalization hypothesis*, according to which, in reading a mixed list of words and non-words, when stimulus quality is low, the system would use a thresholded mode of processing at the letter level, in order to prevent lexicalization errors in reading non-words. This processing mode would stop the cascaded feed-forwarding of the activation from the letter level to the GPC module and to the orthographic lexicon, getting the system to work in a sequential way (Sternberg, 1969). As the stimulus quality lowers, the higher the threshold will be for activation of letter nodes. After that level, the process continues to operate in its usual mode, with parallel activation of lexical representations (for words) and sequential implementation of the GPC rules (for non-words). Thus the effects of stimulus quality and word frequency will be additive. In the case of pure lists of words, the threshold of the letter level is not required, as only lexical representations are involved, so a low level of stimulus quality would interfere more with the activation of low frequency than of high frequency representations. As a consequence, an overadditivity of the frequency effect would appear.

The CDP+ model (Ziegler et al., 2009) offers mechanisms useful to simulate the suggestions made by O’Malley and Besner (2008). In fact, in the CDP+ model, the non-lexical route reaches a threshold, while the lexical route is cascaded. Thus, the observation of additive effects would depend on the strength of the non-lexical route in comparison to the lexical one: in naming a mixed list of words and non-words the lexical route would be de-emphasized, in order to avoid lexicalization of non-words, and in this condition additive effects would appear. Moreover, in the case of a very low stimulus quality, the sensible reduction of the activation in the lexical route would lead to a small word frequency effect, giving rise to an underadditive effect, with high frequency words affected more by low stimulus quality than low frequency words.

Interesting clues for understanding how the context can influence word processing arise from a recent work by Scaltritti et al. (2013). In a naming task in which semantic priming, stimulus quality and frequency effects were assessed, the authors presented both words and non-words. They found an additive effect of stimulus quality and frequency in the case of related primes, but an overadditivity effect in the case of unrelated primes, since low-frequency words were more disrupted by low stimulus quality than high frequency words, as in Borowsky and Besner’s (1993) study. The authors explained these results in terms of the *prime reliance account*, according to which the reliance on prime information is higher in the case of degraded stimuli than in the case of clear stimuli. The prime information is particularly helpful for low-frequency degraded words and this support can compensate for the disruptive effect of low stimulus quality, decreasing the likelihood of an interaction between stimulus quality and frequency. On the contrary, in the case of unrelated primes, the low-frequency degraded words cannot gain advantage from the prime, so they are particularly disrupted in comparison to high-frequency words. In this case, an overadditivity effect is likely to emerge. These results show that the reliance on the prime can be considered a strategy influenced by the list composition: in the case of all unrelated prime-target pairs (Scaltritti et al., 2013: Experiment 2), the information from the prime is skipped and only the expected additivity effect of stimulus quality and frequency is found (see O’Malley and Besner, 2008).

According to the *episodic account* (Bodner and Masson, 2003), reliance on the prime can be varied according to the proportion of trials in which prime and target are semantically related (RP: *relatedness proportion*) and can produce different biases on RTs. If prime reliance is high (high RP), then related-prime targets would speed up, while unrelated-prime targets should be slowed down, due to the potential interference from the prime. Results from a lexical decision task with masked priming (Bodner and Masson, 2003) showed that semantic priming is higher when RP is 0.80 than when RP is 0.20, but no clear inhibition was observed for unrelated-prime targets and this result is inconsistent with an episodic account.

Bodner and Masson (2003), in a further experiment (Experiment 2), found a similar effect of RP, also in the case in which 80% of related primes had different relatedness with the target. In the experiment, high RP condition was made of 20% of semantically related prime-target pairs (e.g., nurse–DOCTOR), the same as low RP condition, and 60% of repetition primes (e.g., doctor–DOCTOR). The authors suggested that their results are neither consistent with the idea of automatic spreading activation within the orthographic lexicon, nor with consciously controlled processes like expectancy and semantic matching, because the prime-target SOA of 45 ms should not be long enough to carry out such processes. These data would suggest that “enhanced reliance on masked prime resources operates in a rather general manner, making use of whatever relation holds between a related prime and target” (p. 650) and indicate the role of prime reliance in creating “a form of episodic resource that can be recruited to assist with target processing” (p. 651).

**FIGURE 5:**
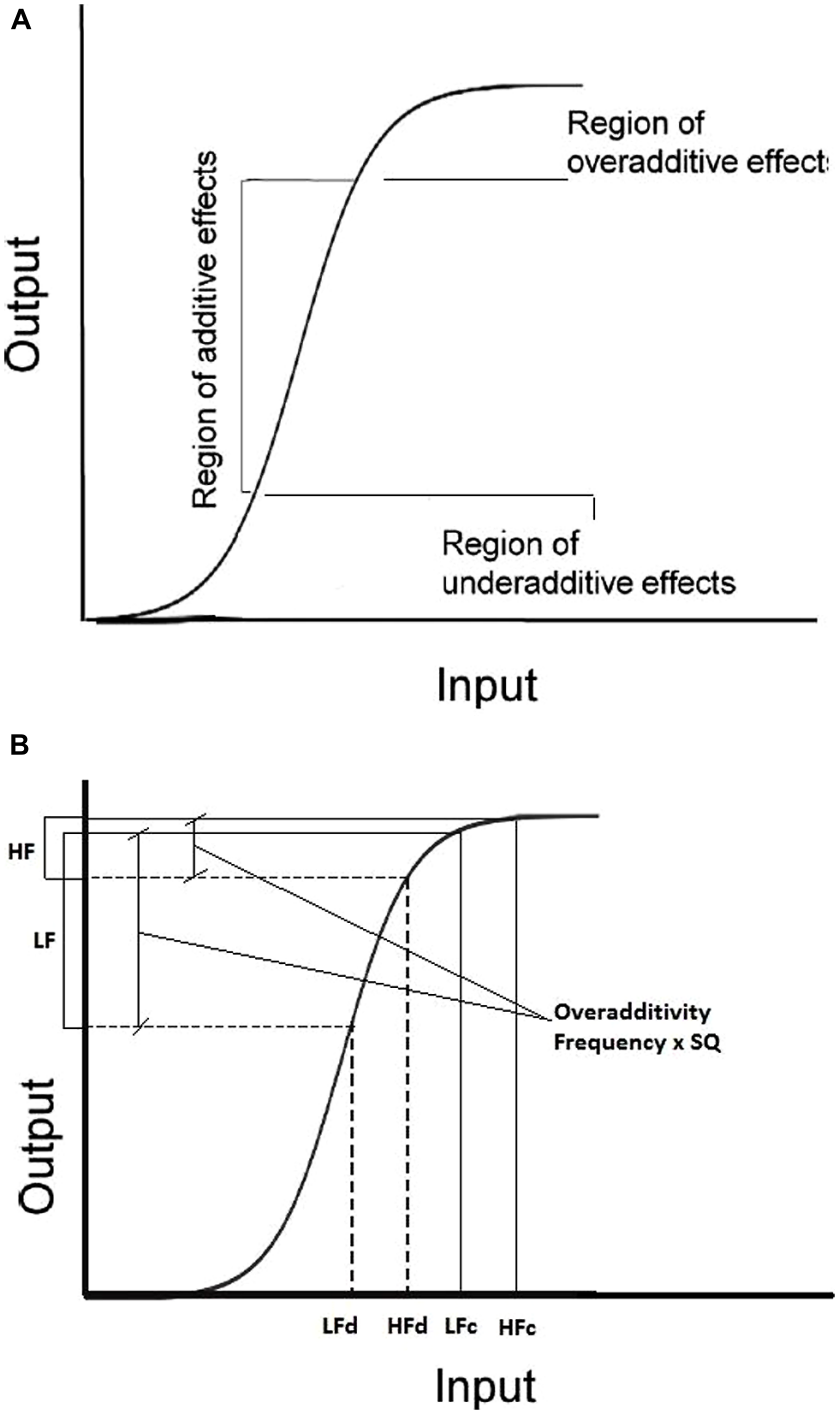
**Representation of the Plaut and Booth (2000) input–output activation function. (A)** regions of the curve corresponding to underadditive, additive, and overadditive effect. **(B)** example of application of the function to data from a 2 × 2 factorial design Frequency (High-Low) × Stimulus Quality (SQ: degraded-clear).

Within the PDP approach, Plaut and Booth (2000, 2006) proposed a sigmoidal function relating input to output, that offers an interesting framework to interpret “how” the previous trials and/or the list context can influence the size of additivity or interaction effects. According to this function (see **Figure 5**), if the input activation values of all target types (e.g., the four points in a 2 × 2 factorial design: high-low frequency, degraded-clear stimulus quality) are in correspondence with the steep section of the curve, there is a quasi-linear increment of the effect size for both conditions, with an additive effect in the output values. If the value of one or more target types is associated with activations corresponding to different sections of the curve, then overadditive (highest part of the curve) or underadditive (lowest part of the curve) effects in output values are expected.

The model of Plaut and Booth (2000) offers an interesting account of different kinds of effects across variables, but it leaves open the question concerning the variables that can determine the change in the level of activation on the input axis. The work of Kinoshita et al. (2011) provides possible suggestions in underscoring the role of recent trials on the processing of a current stimulus. Their model, the adaptation to the statistics of the enviroment (ASE), is based on the results obtained from a linear mixed-effect model analysis (Baayen et al., 2008). This analysis allowed the authors to prove the effect of the previous trial on the processing of the target, both as a main effect and in interaction with other features of the context and of the current stimulus. This model mirrors the time-criterion hypothesis described above, as it assumes that after an easy stimulus, the latency in the next trial will decrease, while after a difficult stimulus, the latency will increase.

An interesting application of an integrated approach of the two models (Plaut and Booth, 2000; Kinoshita et al., 2011) can be found in Masson and Kliegl’s (2013) work. In their experiments, Masson and Kliegl (2013) adopted a semantic priming paradigm, with a prime-target SOA of 200 ms, and varied the stimulus quality. In the ANOVA on aggregated data, they found the usual additivity effect between frequency and stimulus quality, but analyses through the mixed-effect model revealed a completely different pattern of results. The following variables were entered in the model: priming relation (related–unrelated), word frequency (high–low) and stimulus quality (clear-degraded) of the target corresponding to the analyzed RT; the lexical status and the stimulus quality of the last-trial target. All three variables (priming relation, frequency, stimulus quality) characterizing the target were significant and showed a pattern of additivity as expected according to the literature and to the results found with the ANOVA technique.

Additionally, the variables referring to the last-trial target were involved in a significant interaction with the three target variables: if the last-trial target was a degraded non-word, priming was more effective for low-frequency targets and almost nil for high-frequency words, giving rise to an overadditivity effect. This result is consistent with the ASE model, because an extremely difficult item such as a degraded non-word is likely to require more evidence in responding to the next trial. If this increased activation in the input signal is represented in the sigmoid curve proposed by Plaut and Booth (2006), the highest part of the sigmoid curve is involved (see **Figure 5**), thus an overadditivity effect appears. On the contrary, when the last-trial target was a clear word, priming was effective only for high-frequency targets (underadditivity). The underadditive effect in the case of a clear previous word could be explained with the reverse reasoning: “a less demanding experience on trial *n* - 1 might allow the output activation threshold to be lowered, moving the criterion back down the sigmoid function” (p. 906). This shift would produce the observed underadditive effect.

These results proved that recent trial history can exert an important influence on word processing. Considering this component, Masson and Kliegl (2013) claim that the additivity effect between frequency and stimulus quality can also be described as the consequence of two opposite interactions: an overadditivity effect, when the input activation required for responding to the target is high, due to the difficulty of the last-trial target (e.g., degraded non-word), and an underadditivity effect when the last-trial target is easy (e.g., clear word) and the required input activation is low.

O’Malley and Besner (2013) observed that the effects found by Masson and Kliegl (2013) could be “a reflection of decision-level processes specific to the lexical decision task” (p. 1322), so they examined the effect of prior trial history in reading aloud tasks. They found a main effect of prior trial history, but no interactions between this factor and each of the two main factors – stimulus quality and word frequency – was observed. The authors ascribed the effects observed by Masson and Kliegl (2013) to the presence of semantic priming in the lexical decision task, that would promote retrospective processing in a significant way in comparison with other tasks, such as reading aloud.

However, even Masson and Kliegl (2013) failed to find the expected overadditivity effect when the last-trial target was a degraded non-word and the target was not primed. This condition, arguably the most difficult one, gave rise to an underadditivity effect when the stimulus quality was kept constant within a block of trials. The authors suggested that this anomalous outcome could be “the product of a ceiling effect on response time in the slowest condition (low-frequency and degraded target)” (p. 909), that would prevent appreciating a significant change in RTs in comparison to high-frequency words.

Overall, not even the combination of the ASE model (Kinoshita et al., 2011) with the activation function proposed by Plaut and Booth (2000) is able to thoroughly explain all behavioral data on list context effects, but it is a new and interesting approach, that offers some hints for modeling reading processes and for carrying on data analysis in experimental research.

## CONCLUSION

Experimental evidence on list context effects reveals that pronouncing a string of letters is considerably more than an automatic process. In a very simple task like the naming of single items, there are complex interactions among the stimulus properties (psycholinguistic features and stimulus quality), the list context (pure/mixed block), and the properties of the previous stimulus in the list. In addition, data from several languages also show that the orthography-phonology consistency may have a role in determining the usefulness of different strategic settings of the system.

In opaque orthographies, several stimuli are likely to be read correctly only through the lexical pathway (e.g., exception words: PINT, YACHT, etc.), whereas in transparent orthographies most of the words can be read correctly through grapheme-to-phoneme conversion. Hence, skilled readers of opaque orthographies are more likely to be used to shutting down the non-lexical pathway than skilled readers of transparent orthographies. In fact, the non-lexical pathway in transparent orthographies is not very resource-demanding and skilled readers may use it in a highly efficient way. The efficiency in the use of the two pathways develops during literacy acquisition, as some studies on children with and without developmental dyslexia suggest (see Paizi et al., 2011).

How far do current computational models of reading account for the flexibility of the cognitive system and the results of the interaction between orthography and reading processes? The class of dual-route models could be considered more consistent with the de-emphasis hypothesis. In fact, in these models (DRC, CDP) the modification of parameter setting in the non-lexical pathway can be enough to implement list context effects. However, in this framework a new component is required, assumed to operate in two different ways: either choosing which route is to be de-emphasized, or deciding which of the two outputs (from the lexical and from the non-lexical pathway, respectively) is to be taken into account. Moreover, differences in the orthographic consistency of the language can influence the usefulness of de-emphasizing the lexical or the non-lexical route.

The time–criterion hypothesis offers an interpretation of list context effects that is independent of any specific pathway or control mechanism, while introducing the view of reading as a dynamic process, in which the overall level of activation is a function of previous trials. The ASE model, grounded on mixed-effect model statistics, is a recent formal description of the time-criterion hypothesis which, integrated with the Plaut and Booth’s activation function, gives a flexible and probabilistic framework for interpreting additivity and interaction effects. The PDP model, which includes learning mechanisms and is a one-route network, seems to be consistent with this hypothesis. However, in principle, the trial-to-trial changes originating from the easiness/difficulty in processing item in trial *n* -*1* cascading on the processing of the item in trial *n* could be implemented also in a dual-route architecture. To be able to reproduce the dynamic changes induced by list context on stimulus processing, a dual-route model ought to incorporate a thresholded mode of processing (as suggested by O’Malley and Besner, 2008) along with a separate control mechanism modulating route-change procedures (see Reynolds and Besner, 2008) Not even the dynamic approach provided by the ASE model is currently able to account for all the effects found in behavioral data. However, it offers a promising perspective for capturing the peculiarity of human cognition, i.e., flexibility and strategic behavior.

## Conflict of Interest Statement

The authors declare that the research was conducted in the absence of any commercial or financial relationships that could be construed as a potential conflict of interest.

## Footnotes

1 Orthographic neighbors of a string of letters have been operationalized by Coltheart et al. (1977) as the words that can be obtained by changing one letter and preserving the positions of the other letters. For example, neighbors of WORD are LORD, WARD, WORK.
